# Supplementary material for: Feedback regulation of cytoneme-mediated transport shapes a tissue-specific FGF morphogen gradient
Source: eLife. 2018 Oct 17;7:e38137. doi: 10.7554/eLife.38137 (PMC6224196; doi:10.7554/eLife.38137)
Supplement: Figure 5—source data 2. [file elife-38137-fig5-data2.docx]

**Table. Number of ASP cytonemes oriented in different directions from *wt* clones (Fig. 5C)**

| **Distal clone_WT (1-6 cell distance from tip)** | | | | | | | | |
| --- | --- | --- | --- | --- | --- | --- | --- | --- |
| **Directional area** | **# cytonemes with length 0-15μm** | | | | | | | **Average** |
| 0-30 | 3 | 1 | 3 | 3 | 1 | 4 | 2 | 2.4 |
| 30-60 | 0 | 0 | 0 | 0 | 0 | 0 | 0 | 0 |
| 60-90 | 0 | 0 | 0 | 5 | 0 | 0 | 0 | 0.7 |
| 90-120 | 0 | 0 | 0 | 3 | 0 | 0 | 0 | 0.4 |
| 120-150 | 0 | 0 | 0 | 0 | 0 | 0 | 0 | 0 |
| 150-180 | 0 | 0 | 0 | 1 | 0 | 0 | 0 | 0.1 |
| 180-210 | 0 | 0 | 0 | 1 | 0 | 0 | 0 | 0.1 |
| 210-240 | 0 | 0 | 0 | 1 | 0 | 0 | 2 | 0.4 |
| 240-270 | 0 | 0 | 0 | 1 | 0 | 2 | 0 | 0.4 |
| 270-300 | 0 | 0 | 0 | 0 | 0 | 4 | 0 | 0.6 |
| 300-330 | 0 | 0 | 0 | 0 | 0 | 0 | 0 | 0 |
| 330-360 | 2 | 1 | 5 | 8 | 0 | 3 | 1 | 2.8 |
| Total | 5 | 2 | 8 | 23 | 1 | 13 | 5 | 8.1 |
| **Directional area** | **# cytonemes with length 15-30μm** | | | | | | | **Average** |
| 0-30 | 3 | 2 | 2 | 5 | 5 | 2 | 2 | 3 |
| 30-60 | 0 | 0 | 0 | 0 | 0 | 0 | 0 | 0 |
| 60-90 | 0 | 0 | 0 | 0 | 0 | 0 | 0 | 0 |
| 90-120 | 0 | 0 | 0 | 0 | 0 | 0 | 0 | 0 |
| 120-150 | 0 | 0 | 0 | 0 | 0 | 0 | 0 | 0 |
| 150-180 | 0 | 0 | 0 | 0 | 0 | 0 | 0 | 0 |
| 180-210 | 0 | 0 | 0 | 0 | 0 | 0 | 0 | 0 |
| 210-240 | 0 | 0 | 0 | 0 | 0 | 0 | 0 | 0 |
| 240-270 | 0 | 0 | 0 | 0 | 0 | 0 | 0 | 0 |
| 270-300 | 0 | 0 | 0 | 0 | 0 | 0 | 0 | 0 |
| 300-330 | 1 | 0 | 0 | 0 | 0 | 0 | 0 | 0.1 |
| 330-360 | 3 | 2 | 1 | 5 | 5 | 1 | 1 | 2.6 |
| Total | 7 | 4 | 3 | 10 | 10 | 3 | 3 | 5.7 |
| **Directional area** | **# cytonemes with length >30μm** | | | | | | | **Average** |
| 0-30 | 0 | 0 | 0 | 0 | 2 | 0 | 0 | 0.3 |
| 30-60 | 0 | 0 | 0 | 0 | 0 | 0 | 0 | 0 |
| 60-90 | 0 | 0 | 0 | 0 | 0 | 0 | 0 | 0 |
| 90-120 | 0 | 0 | 0 | 0 | 0 | 0 | 0 | 0 |
| 120-150 | 0 | 0 | 0 | 0 | 0 | 0 | 0 | 0 |
| 150-180 | 0 | 0 | 0 | 0 | 0 | 0 | 0 | 0 |
| 180-210 | 0 | 0 | 0 | 0 | 0 | 0 | 0 | 0 |
| 210-240 | 0 | 0 | 0 | 0 | 0 | 0 | 0 | 0 |
| 240-270 | 0 | 0 | 0 | 0 | 0 | 0 | 0 | 0 |
| 270-300 | 0 | 0 | 0 | 0 | 0 | 0 | 0 | 0 |
| 300-330 | 0 | 0 | 0 | 0 | 0 | 0 | 0 | 0 |
| 330-360 | 0 | 0 | 0 | 0 | 0 | 0 | 0 | 0 |
| Total | 0 | 0 | 0 | 0 | 2 | 0 | 0 | 0.3 |

| **Proximal clone_*WT* (7 cell distance onward from tip)** | | | | | | | | | | | | |
| --- | --- | --- | --- | --- | --- | --- | --- | --- | --- | --- | --- | --- |
| **Directional area** | **# cytonemes with length 0-15μm** | | | | | | | | | | | **Average** |
| 0-30 | 2 | 2 | 1 | 1 | 1 | 4 | 1 | 1 | 1 | 4 | 2 | 1.8 |
| 30-60 | 0 | 0 | 2 | 2 | 1 | 0 | 1 | 0 | 5 | 1 | 0 | 1.1 |
| 60-90 | 3 | 5 | 2 | 3 | 0 | 3 | 0 | 5 | 5 | 8 | 0 | 3.1 |
| 90-120 | 0 | 6 | 3 | 3 | 2 | 0 | 1 | 0 | 3 | 2 | 0 | 1.8 |
| 120-150 | 1 | 0 | 2 | 1 | 3 | 1 | 0 | 5 | 0 | 3 | 0 | 1.4 |
| 150-180 | 2 | 1 | 3 | 8 | 0 | 2 | 1 | 0 | 1 | 1 | 0 | 1.7 |
| 180-210 | 0 | 0 | 2 | 4 | 0 | 2 | 1 | 0 | 2 | 0 | 0 | 1 |
| 210-240 | 0 | 0 | 0 | 3 | 0 | 1 | 1 | 0 | 0 | 0 | 0 | 0.4 |
| 240-270 | 0 | 3 | 2 | 5 | 0 | 2 | 1 | 0 | 0 | 0 | 0 | 1.2 |
| 270-300 | 0 | 2 | 1 | 7 | 0 | 5 | 2 | 2 | 1 | 1 | 0 | 1.9 |
| 300-330 | 5 | 1 | 0 | 1 | 1 | 3 | 0 | 1 | 2 | 0 | 0 | 1.3 |
| 330-360 | 7 | 2 | 1 | 1 | 2 | 3 | 4 | 5 | 2 | 1 | 2 | 2.7 |
| Total | 20 | 22 | 19 | 39 | 10 | 26 | 13 | 19 | 22 | 21 | 4 | 19.5 |
| **Directional area** | **# cytonemes with length 15-30μm** | | | | | | | | | | | **Average** |
| 0-30 | 0 | 0 | 0 | 0 | 0 | 0 | 0 | 0 | 1 | 0 | 0 | 0.1 |
| 30-60 | 0 | 0 | 0 | 0 | 0 | 0 | 0 | 0 | 5 | 1 | 0 | 0.5 |
| 60-90 | 0 | 0 | 0 | 0 | 0 | 0 | 0 | 0 | 2 | 0 | 0 | 0.2 |
| 90-120 | 0 | 0 | 0 | 0 | 0 | 0 | 0 | 0 | 0 | 0 | 0 | 0 |
| 120-150 | 0 | 0 | 0 | 0 | 0 | 0 | 0 | 0 | 0 | 0 | 0 | 0 |
| 150-180 | 0 | 0 | 0 | 0 | 0 | 0 | 0 | 0 | 1 | 0 | 0 | 0.1 |
| 180-210 | 0 | 0 | 0 | 0 | 0 | 0 | 0 | 0 | 0 | 0 | 0 | 0 |
| 210-240 | 0 | 0 | 0 | 0 | 0 | 0 | 0 | 0 | 0 | 0 | 0 | 0 |
| 240-270 | 0 | 0 | 0 | 0 | 0 | 0 | 0 | 0 | 0 | 0 | 0 | 0 |
| 270-300 | 0 | 0 | 0 | 0 | 0 | 0 | 0 | 0 | 0 | 0 | 0 | 0 |
| 300-330 | 0 | 0 | 0 | 0 | 0 | 0 | 0 | 0 | 0 | 0 | 0 | 0 |
| 330-360 | 0 | 0 | 0 | 0 | 0 | 0 | 0 | 0 | 0 | 0 | 0 | 0 |
| Total | 0 | 0 | 0 | 0 | 0 | 0 | 0 | 0 | 9 | 1 | 0 | 0.9 |
| **Directional area** | **# cytonemes with length >30μm** | | | | | | | | | | | **Average** |
| 0-30 | 0 | 0 | 0 | 0 | 0 | 0 | 0 | 0 | 0 | 0 | 0 | 0 |
| 30-60 | 0 | 0 | 0 | 0 | 0 | 0 | 0 | 0 | 0 | 0 | 0 | 0 |
| 60-90 | 0 | 0 | 0 | 0 | 0 | 0 | 0 | 0 | 0 | 0 | 0 | 0 |
| 90-120 | 0 | 0 | 0 | 0 | 0 | 0 | 0 | 0 | 0 | 0 | 0 | 0 |
| 120-150 | 0 | 0 | 0 | 0 | 0 | 0 | 0 | 0 | 0 | 0 | 0 | 0 |
| 150-180 | 0 | 0 | 0 | 0 | 0 | 0 | 0 | 0 | 0 | 0 | 0 | 0 |
| 180-210 | 0 | 0 | 0 | 0 | 0 | 0 | 0 | 0 | 0 | 0 | 0 | 0 |
| 210-240 | 0 | 0 | 0 | 0 | 0 | 0 | 0 | 0 | 0 | 0 | 0 | 0 |
| 240-270 | 0 | 0 | 0 | 0 | 0 | 0 | 0 | 0 | 0 | 0 | 0 | 0 |
| 270-300 | 0 | 0 | 0 | 0 | 0 | 0 | 0 | 0 | 0 | 0 | 0 | 0 |
| 300-330 | 0 | 0 | 0 | 0 | 0 | 0 | 0 | 0 | 0 | 0 | 0 | 0 |
| 330-360 | 0 | 0 | 0 | 0 | 0 | 0 | 0 | 0 | 0 | 0 | 0 | 0 |
| Total | 0 | 0 | 0 | 0 | 0 | 0 | 0 | 0 | 0 | 0 | 0 | 0 |

Note: The clone size varies from 1 to 4 cells and there is no significant correlation in the number of cytonemes and clone size within 1-4 cells limit. The directional distribution of cytonemes from each clone was presented in every 30 degree range. The cytoneme counting was grouped by length into 0-15μm, 15-30μm, and >30μm.
