# Supplementary material for: Feedback regulation of cytoneme-mediated transport shapes a tissue-specific FGF morphogen gradient
Source: eLife. 2018 Oct 17;7:e38137. doi: 10.7554/eLife.38137 (PMC6224196; doi:10.7554/eLife.38137)
Supplement: Figure 7—source data 1. [file elife-38137-fig7-data1.docx]

**Number of ASP cytonemes oriented in different directions from various mutant clones.**

A. Cytoneme counting from *pnt-P1* GOF clones (Fig. 7C’)

| **Distal clone_PntP1-GOF (1-6 cell distance from tip)** | | | | | | | | | |
| --- | --- | --- | --- | --- | --- | --- | --- | --- | --- |
| **Directional area** | **# cytonemes with length 0-15μm** | | | | | | | | **Average** |
| 0-30 | 0 | 0 | 0 | 0 | 3 | 0 | 0 | 0 | 0.375 |
| 30-60 | 0 | 0 | 0 | 0 | 0 | 0 | 0 | 0 | 0 |
| 60-90 | 0 | 0 | 0 | 0 | 0 | 0 | 0 | 0 | 0 |
| 90-120 | 0 | 0 | 0 | 0 | 0 | 0 | 0 | 0 | 0 |
| 120-150 | 0 | 0 | 0 | 1 | 0 | 0 | 0 | 0 | 0.125 |
| 150-180 | 0 | 0 | 0 | 0 | 0 | 0 | 0 | 0 | 0 |
| 180-210 | 1 | 1 | 0 | 0 | 0 | 0 | 0 | 0 | 0.25 |
| 210-240 | 0 | 0 | 0 | 0 | 0 | 0 | 0 | 0 | 0 |
| 240-270 | 0 | 0 | 0 | 0 | 0 | 0 | 0 | 0 | 0 |
| 270-300 | 0 | 0 | 0 | 0 | 0 | 0 | 2 | 0 | 0.25 |
| 300-330 | 0 | 0 | 0 | 0 | 1 | 0 | 0 | 0 | 0.125 |
| 330-360 | 1 | 3 | 0 | 0 | 2 | 0 | 0 | 0 | 0.75 |
| Total | 2 | 4 | 0 | 1 | 6 | 0 | 2 | 0 | 1.875 |
| **Directional area** | **# cytonemes with length 15-30μm** | | | | | | | | **Average** |
| 0-30 | 4 | 3 | 2 | 1 | 3 | 1 | 2 | 5 | 2.625 |
| 30-60 | 0 | 0 | 0 | 0 | 0 | 0 | 0 | 0 | 0 |
| 60-90 | 0 | 0 | 0 | 0 | 0 | 0 | 0 | 0 | 0 |
| 90-120 | 0 | 0 | 0 | 0 | 0 | 0 | 0 | 0 | 0 |
| 120-150 | 0 | 0 | 0 | 0 | 0 | 0 | 0 | 0 | 0 |
| 150-180 | 0 | 0 | 0 | 0 | 0 | 0 | 0 | 0 | 0 |
| 180-210 | 0 | 0 | 0 | 0 | 0 | 0 | 0 | 0 | 0 |
| 210-240 | 0 | 0 | 0 | 0 | 0 | 0 | 0 | 0 | 0 |
| 240-270 | 0 | 0 | 0 | 0 | 0 | 0 | 0 | 0 | 0 |
| 270-300 | 0 | 0 | 0 | 0 | 0 | 0 | 0 | 0 | 0 |
| 300-330 | 0 | 0 | 0 | 0 | 0 | 0 | 0 | 0 | 0 |
| 330-360 | 4 | 2 | 1 | 0 | 3 | 3 | 2 | 4 | 2.375 |
| Total | 8 | 5 | 3 | 1 | 6 | 4 | 4 | 9 | 5 |
| **Directional area** | **# cytonemes with length >30μm** | | | | | | | | **Average** |
| 0-30 | 1 | 3 | 1 | 2 | 1 | 3 | 1 | 2 | 1.75 |
| 30-60 | 0 | 0 | 0 | 0 | 0 | 0 | 0 | 0 | 0 |
| 60-90 | 0 | 0 | 0 | 0 | 0 | 0 | 0 | 0 | 0 |
| 90-120 | 0 | 0 | 0 | 0 | 0 | 0 | 0 | 0 | 0 |
| 120-150 | 0 | 0 | 0 | 0 | 0 | 0 | 0 | 0 | 0 |
| 150-180 | 0 | 0 | 0 | 0 | 0 | 0 | 0 | 0 | 0 |
| 180-210 | 0 | 0 | 0 | 0 | 0 | 0 | 0 | 0 | 0 |
| 210-240 | 0 | 0 | 0 | 0 | 0 | 0 | 0 | 0 | 0 |
| 240-270 | 0 | 0 | 0 | 0 | 0 | 0 | 0 | 0 | 0 |
| 270-300 | 0 | 0 | 0 | 0 | 0 | 0 | 0 | 0 | 0 |
| 300-330 | 0 | 0 | 0 | 0 | 0 | 0 | 0 | 0 | 0 |
| 330-360 | 0 | 0 | 2 | 1 | 0 | 2 | 0 | 1 | 0.75 |
| Total | 1 | 3 | 3 | 3 | 1 | 5 | 1 | 3 | 2.5 |

| **Proximal clone_PntP1-GOF (7 cell distance onward from tip)** | | | | | | |
| --- | --- | --- | --- | --- | --- | --- |
| **Directional area** | **# cytonemes with length 0-15μm** | | | | | **Average** |
| 0-30 | 0 | 0 | 3 | 2 | 2 | 1.4 |
| 30-60 | 0 | 0 | 0 | 0 | 0 | 0 |
| 60-90 | 0 | 1 | 0 | 0 | 0 | 0.2 |
| 90-120 | 0 | 0 | 0 | 0 | 0 | 0 |
| 120-150 | 0 | 0 | 0 | 0 | 0 | 0 |
| 150-180 | 0 | 0 | 0 | 0 | 0 | 0 |
| 180-210 | 0 | 0 | 0 | 0 | 0 | 0 |
| 210-240 | 0 | 0 | 0 | 0 | 0 | 0 |
| 240-270 | 0 | 0 | 0 | 0 | 0 | 0 |
| 270-300 | 0 | 0 | 0 | 0 | 0 | 0 |
| 300-330 | 0 | 2 | 0 | 2 | 0 | 0.8 |
| 330-360 | 0 | 0 | 0 | 2 | 2 | 0.8 |
| Total | 0 | 3 | 3 | 6 | 4 | 3.2 |
| **Directional area** | **# cytonemes with length 15-30μm** | | | | | **Average** |
| 0-30 | 3 | 0 | 0 | 2 | 0 | 1 |
| 30-60 | 0 | 0 | 0 | 0 | 0 | 0 |
| 60-90 | 0 | 0 | 0 | 0 | 0 | 0 |
| 90-120 | 0 | 0 | 0 | 0 | 0 | 0 |
| 120-150 | 0 | 0 | 0 | 0 | 0 | 0 |
| 150-180 | 0 | 0 | 0 | 0 | 0 | 0 |
| 180-210 | 0 | 0 | 0 | 0 | 0 | 0 |
| 210-240 | 0 | 0 | 0 | 0 | 0 | 0 |
| 240-270 | 0 | 0 | 0 | 0 | 0 | 0 |
| 270-300 | 0 | 0 | 0 | 0 | 0 | 0 |
| 300-330 | 0 | 0 | 0 | 0 | 0 | 0 |
| 330-360 | 0 | 0 | 0 | 4 | 0 | 0.8 |
| Total | 3 | 0 | 0 | 6 | 0 | 1.8 |
| **Directional area** | **# cytonemes with length >30μm** | | | | | **Average** |
| 0-30 | 2 | 1 | 2 | 0 | 2 | 1.4 |
| 30-60 | 0 | 0 | 0 | 0 | 0 | 0 |
| 60-90 | 0 | 0 | 0 | 0 | 0 | 0 |
| 90-120 | 0 | 0 | 0 | 0 | 0 | 0 |
| 120-150 | 0 | 0 | 0 | 0 | 0 | 0 |
| 150-180 | 0 | 0 | 0 | 0 | 0 | 0 |
| 180-210 | 0 | 0 | 0 | 0 | 0 | 0 |
| 210-240 | 0 | 0 | 0 | 0 | 0 | 0 |
| 240-270 | 0 | 0 | 0 | 0 | 0 | 0 |
| 270-300 | 0 | 0 | 0 | 0 | 0 | 0 |
| 300-330 | 0 | 0 | 0 | 0 | 0 | 0 |
| 330-360 | 2 | 2 | 4 | 0 | 2 | 2 |
| Total | 4 | 3 | 6 | 0 | 4 | 3.4 |

B. Cytoneme counting from *pntRNAi* clones (Fig. 7D’)

| **Distal clone_*pntRNAi* (1-6 cell distance from tip)** | | | | | | | | | | | | |
| --- | --- | --- | --- | --- | --- | --- | --- | --- | --- | --- | --- | --- |
| **Directional area** | **# cytonemes with length 0-15μm** | | | | | | | | | | | **Average** |
| 0-30 | 1 | 0 | 0 | 2 | 2 | 2 | 2 | 2 | 0 | 3 | 2 | 1.45 |
| 30-60 | 0 | 0 | 0 | 2 | 1 | 1 | 2 | 2 | 0 | 4 | 2 | 1.27 |
| 60-90 | 0 | 0 | 0 | 1 | 2 | 1 | 0 | 5 | 0 | 1 | 1 | 1.00 |
| 90-120 | 0 | 0 | 0 | 2 | 0 | 1 | 1 | 2 | 0 | 1 | 1 | 0.73 |
| 120-150 | 0 | 0 | 0 | 0 | 0 | 0 | 0 | 0 | 0 | 1 | 0 | 0.09 |
| 150-180 | 0 | 0 | 0 | 0 | 0 | 0 | 0 | 0 | 0 | 0 | 0 | 0.00 |
| 180-210 | 0 | 0 | 0 | 0 | 0 | 0 | 0 | 2 | 0 | 2 | 0 | 0.36 |
| 210-240 | 0 | 0 | 0 | 0 | 0 | 0 | 0 | 0 | 0 | 0 | 0 | 0.00 |
| 240-270 | 0 | 0 | 0 | 0 | 0 | 1 | 1 | 1 | 0 | 3 | 0 | 0.55 |
| 270-300 | 0 | 0 | 0 | 1 | 1 | 0 | 1 | 2 | 4 | 3 | 1 | 1.18 |
| 300-330 | 0 | 0 | 1 | 0 | 6 | 2 | 1 | 0 | 2 | 3 | 0 | 1.36 |
| 330-360 | 1 | 0 | 1 | 3 | 3 | 2 | 2 | 2 | 4 | 3 | 4 | 2.27 |
| Total | 2 | 0 | 2 | 11 | 15 | 10 | 10 | 18 | 10 | 24 | 11 | 10.27 |
| **Directional area** | **# cytonemes with length 15-30μm** | | | | | | | | | | | **Average** |
| 0-30 | 1 | 0 | 2 | 0 | 1 | 0 | 1 | 0 | 1 | 1 | 2 | 0.82 |
| 30-60 | 0 | 0 | 0 | 0 | 0 | 0 | 0 | 0 | 0 | 1 | 0 | 0.09 |
| 60-90 | 0 | 0 | 0 | 0 | 0 | 0 | 0 | 0 | 0 | 0 | 0 | 0.00 |
| 90-120 | 0 | 0 | 0 | 0 | 0 | 0 | 0 | 0 | 0 | 0 | 0 | 0.00 |
| 120-150 | 0 | 0 | 0 | 0 | 0 | 0 | 0 | 0 | 0 | 0 | 0 | 0.00 |
| 150-180 | 0 | 0 | 0 | 0 | 0 | 0 | 0 | 0 | 0 | 0 | 0 | 0.00 |
| 180-210 | 0 | 0 | 0 | 0 | 0 | 0 | 0 | 0 | 0 | 0 | 0 | 0.00 |
| 210-240 | 0 | 0 | 0 | 0 | 0 | 0 | 0 | 0 | 0 | 0 | 0 | 0.00 |
| 240-270 | 0 | 0 | 0 | 0 | 0 | 0 | 0 | 0 | 0 | 0 | 0 | 0.00 |
| 270-300 | 0 | 0 | 0 | 0 | 0 | 0 | 0 | 0 | 0 | 0 | 0 | 0.00 |
| 300-330 | 0 | 0 | 0 | 0 | 0 | 0 | 0 | 0 | 0 | 0 | 0 | 0.00 |
| 330-360 | 0 | 1 | 0 | 0 | 0 | 0 | 0 | 0 | 2 | 1 | 0 | 0.36 |
| Total | 1 | 1 | 2 | 0 | 1 | 0 | 1 | 0 | 3 | 3 | 2 | 1.27 |
| **Directional area** | **# cytonemes with length >30μm** | | | | | | | | | | | **Average** |
| 0-30 | 0 | 0 | 0 | 0 | 0 | 0 | 0 | 0 | 0 | 0 | 0 | 0 |
| 30-60 | 0 | 0 | 0 | 0 | 0 | 0 | 0 | 0 | 0 | 0 | 0 | 0 |
| 60-90 | 0 | 0 | 0 | 0 | 0 | 0 | 0 | 0 | 0 | 0 | 0 | 0 |
| 90-120 | 0 | 0 | 0 | 0 | 0 | 0 | 0 | 0 | 0 | 0 | 0 | 0 |
| 120-150 | 0 | 0 | 0 | 0 | 0 | 0 | 0 | 0 | 0 | 0 | 0 | 0 |
| 150-180 | 0 | 0 | 0 | 0 | 0 | 0 | 0 | 0 | 0 | 0 | 0 | 0 |
| 180-210 | 0 | 0 | 0 | 0 | 0 | 0 | 0 | 0 | 0 | 0 | 0 | 0 |
| 210-240 | 0 | 0 | 0 | 0 | 0 | 0 | 0 | 0 | 0 | 0 | 0 | 0 |
| 240-270 | 0 | 0 | 0 | 0 | 0 | 0 | 0 | 0 | 0 | 0 | 0 | 0 |
| 270-300 | 0 | 0 | 0 | 0 | 0 | 0 | 0 | 0 | 0 | 0 | 0 | 0 |
| 300-330 | 0 | 0 | 0 | 0 | 0 | 0 | 0 | 0 | 0 | 0 | 0 | 0 |
| 330-360 | 0 | 0 | 0 | 0 | 0 | 0 | 0 | 0 | 0 | 0 | 0 | 0 |
| Total | 0 | 0 | 0 | 0 | 0 | 0 | 0 | 0 | 0 | 0 | 0 | 0 |

| **Proximal clone_*pntRNAi* (7 cell distance onward from tip)** | | | | | | | |
| --- | --- | --- | --- | --- | --- | --- | --- |
| **Directional area** | **# cytonemes with length 0-15μm** | | | | | | **Average** |
| 0-30 | 1 | 3 | 3 | 1 | 1 | 1 | 1.67 |
| 30-60 | 2 | 3 | 2 | 0 | 0 | 0 | 1.17 |
| 60-90 | 1 | 5 | 1 | 2 | 2 | 0 | 1.83 |
| 90-120 | 2 | 5 | 0 | 1 | 7 | 0 | 2.50 |
| 120-150 | 0 | 3 | 1 | 0 | 1 | 1 | 1.00 |
| 150-180 | 0 | 0 | 0 | 2 | 3 | 2 | 1.17 |
| 180-210 | 0 | 0 | 0 | 0 | 0 | 1 | 0.17 |
| 210-240 | 0 | 0 | 0 | 0 | 0 | 0 | 0.00 |
| 240-270 | 2 | 0 | 1 | 3 | 0 | 2 | 1.33 |
| 270-300 | 3 | 0 | 4 | 3 | 0 | 0 | 1.67 |
| 300-330 | 3 | 0 | 3 | 1 | 0 | 1 | 1.33 |
| 330-360 | 5 | 1 | 2 | 2 | 0 | 1 | 1.83 |
| Total | 19 | 20 | 17 | 15 | 14 | 9 | 15.67 |
| **Directional area** | **# cytonemes with length 15-30μm** | | | | | | **Average** |
| 0-30 | 3 | 0 | 0 | 0 | 0 | 0 | 0.50 |
| 30-60 | 1 | 0 | 0 | 0 | 0 | 0 | 0.17 |
| 60-90 | 0 | 0 | 0 | 0 | 0 | 0 | 0.00 |
| 90-120 | 0 | 0 | 0 | 0 | 0 | 0 | 0.00 |
| 120-150 | 0 | 0 | 0 | 0 | 0 | 0 | 0.00 |
| 150-180 | 0 | 0 | 0 | 0 | 0 | 0 | 0.00 |
| 180-210 | 0 | 0 | 0 | 0 | 0 | 0 | 0.00 |
| 210-240 | 0 | 0 | 0 | 0 | 0 | 0 | 0.00 |
| 240-270 | 0 | 0 | 0 | 0 | 0 | 0 | 0.00 |
| 270-300 | 0 | 0 | 0 | 0 | 0 | 0 | 0.00 |
| 300-330 | 0 | 0 | 0 | 0 | 0 | 0 | 0.00 |
| 330-360 | 0 | 0 | 0 | 0 | 0 | 0 | 0.00 |
| Total | 4 | 0 | 0 | 0 | 0 | 0 | 0.67 |
| **Directional area** | **# cytonemes with length >30μm** | | | | | | **Average** |
| 0-30 | 0 | 0 | 0 | 0 | 0 | 0 | 0 |
| 30-60 | 0 | 0 | 0 | 0 | 0 | 0 | 0 |
| 60-90 | 0 | 0 | 0 | 0 | 0 | 0 | 0 |
| 90-120 | 0 | 0 | 0 | 0 | 0 | 0 | 0 |
| 120-150 | 0 | 0 | 0 | 0 | 0 | 0 | 0 |
| 150-180 | 0 | 0 | 0 | 0 | 0 | 0 | 0 |
| 180-210 | 0 | 0 | 0 | 0 | 0 | 0 | 0 |
| 210-240 | 0 | 0 | 0 | 0 | 0 | 0 | 0 |
| 240-270 | 0 | 0 | 0 | 0 | 0 | 0 | 0 |
| 270-300 | 0 | 0 | 0 | 0 | 0 | 0 | 0 |
| 300-330 | 0 | 0 | 0 | 0 | 0 | 0 | 0 |
| 330-360 | 0 | 0 | 0 | 0 | 0 | 0 | 0 |
| Total | 0 | 0 | 0 | 0 | 0 | 0 | 0 |

C. Cytoneme counting from Cut GOF clones (Fig. 7A’)

| **Distal clone_Cut-GOF (1-6 cell distance from tip)** | | | | | | | | | | | | | |
| --- | --- | --- | --- | --- | --- | --- | --- | --- | --- | --- | --- | --- | --- |
| **Directional area** | **# cytonemes with length 0-15μm** | | | | | | | | | | | | **Average** |
| 0-30 | 5 | 2 | 2 | 2 | 0 | 2 | 2 | 1 | 1 | 0 | 2 | 2 | 1.8 |
| 30-60 | 2 | 1 | 0 | 0 | 0 | 1 | 0 | 0 | 0 | 0 | 0 | 0 | 0.3 |
| 60-90 | 1 | 2 | 0 | 0 | 0 | 3 | 0 | 0 | 1 | 0 | 0 | 0 | 0.6 |
| 90-120 | 0 | 0 | 0 | 0 | 0 | 1 | 0 | 2 | 1 | 2 | 2 | 0 | 0.7 |
| 120-150 | 0 | 0 | 0 | 0 | 0 | 0 | 0 | 0 | 0 | 0 | 0 | 0 | 0.0 |
| 150-180 | 0 | 0 | 0 | 0 | 0 | 0 | 0 | 0 | 0 | 0 | 0 | 0 | 0.0 |
| 180-210 | 2 | 0 | 0 | 0 | 0 | 0 | 0 | 0 | 0 | 0 | 3 | 0 | 0.4 |
| 210-240 | 3 | 0 | 0 | 0 | 0 | 1 | 0 | 0 | 0 | 0 | 1 | 0 | 0.4 |
| 240-270 | 3 | 0 | 0 | 0 | 0 | 1 | 0 | 0 | 1 | 0 | 0 | 0 | 0.4 |
| 270-300 | 0 | 0 | 0 | 0 | 0 | 0 | 0 | 0 | 1 | 0 | 0 | 0 | 0.1 |
| 300-330 | 3 | 1 | 1 | 0 | 0 | 2 | 0 | 0 | 0 | 0 | 0 | 0 | 0.6 |
| 330-360 | 0 | 3 | 1 | 0 | 1 | 0 | 1 | 1 | 4 | 0 | 0 | 2 | 1.1 |
| Total | 19 | 9 | 4 | 2 | 1 | 11 | 3 | 4 | 9 | 2 | 8 | 4 | 6.3 |
| **Directional area** | **# cytonemes with length 15-30μm** | | | | | | | | | | | | **Average** |
| 0-30 | 1 | 1 | 0 | 0 | 0 | 0 | 0 | 1 | 1 | 0 | 1 | 1 | 0.5 |
| 30-60 | 0 | 0 | 0 | 0 | 0 | 0 | 0 | 0 | 0 | 0 | 0 | 0 | 0.0 |
| 60-90 | 0 | 0 | 0 | 0 | 0 | 0 | 0 | 0 | 0 | 0 | 0 | 0 | 0.0 |
| 90-120 | 0 | 0 | 0 | 0 | 0 | 0 | 0 | 0 | 0 | 0 | 0 | 0 | 0.0 |
| 120-150 | 0 | 0 | 0 | 0 | 0 | 0 | 0 | 0 | 0 | 0 | 0 | 0 | 0.0 |
| 150-180 | 0 | 0 | 0 | 0 | 0 | 0 | 0 | 0 | 0 | 0 | 0 | 0 | 0.0 |
| 180-210 | 0 | 0 | 0 | 0 | 0 | 0 | 0 | 0 | 0 | 0 | 0 | 0 | 0.0 |
| 210-240 | 1 | 1 | 0 | 0 | 0 | 0 | 0 | 0 | 0 | 0 | 0 | 0 | 0.2 |
| 240-270 | 0 | 0 | 0 | 0 | 0 | 0 | 0 | 0 | 0 | 0 | 0 | 0 | 0.0 |
| 270-300 | 0 | 0 | 0 | 0 | 0 | 0 | 0 | 0 | 0 | 0 | 0 | 0 | 0.0 |
| 300-330 | 0 | 0 | 0 | 0 | 0 | 0 | 0 | 0 | 0 | 0 | 0 | 0 | 0.0 |
| 330-360 | 1 | 1 | 0 | 0 | 0 | 0 | 0 | 0 | 0 | 0 | 1 | 0 | 0.3 |
| Total | 3 | 3 | 0 | 0 | 0 | 0 | 0 | 1 | 1 | 0 | 2 | 1 | 0.9 |
| **Directional area** | **# cytonemes with length >30μm** | | | | | | | | | | | | **Average** |
| 0-30 | 0 | 0 | 0 | 0 | 0 | 0 | 0 | 0 | 1 | 0 | 0 | 0 | 0.1 |
| 30-60 | 0 | 0 | 0 | 0 | 0 | 0 | 0 | 0 | 0 | 0 | 0 | 0 | 0.0 |
| 60-90 | 0 | 0 | 0 | 0 | 0 | 0 | 0 | 0 | 0 | 0 | 0 | 0 | 0.0 |
| 90-120 | 0 | 0 | 0 | 0 | 0 | 0 | 0 | 0 | 0 | 0 | 0 | 0 | 0.0 |
| 120-150 | 0 | 0 | 0 | 0 | 0 | 0 | 0 | 0 | 0 | 0 | 0 | 0 | 0.0 |
| 150-180 | 0 | 0 | 0 | 0 | 0 | 0 | 0 | 0 | 0 | 0 | 0 | 0 | 0.0 |
| 180-210 | 0 | 0 | 0 | 0 | 0 | 0 | 0 | 0 | 0 | 0 | 0 | 0 | 0.0 |
| 210-240 | 0 | 0 | 0 | 0 | 0 | 0 | 0 | 0 | 0 | 0 | 0 | 0 | 0.0 |
| 240-270 | 0 | 0 | 0 | 0 | 0 | 0 | 0 | 0 | 0 | 0 | 0 | 0 | 0.0 |
| 270-300 | 0 | 0 | 0 | 0 | 0 | 0 | 0 | 0 | 0 | 0 | 0 | 0 | 0.0 |
| 300-330 | 0 | 0 | 0 | 0 | 0 | 0 | 0 | 0 | 0 | 0 | 0 | 0 | 0.0 |
| 330-360 | 0 | 0 | 0 | 0 | 0 | 0 | 0 | 0 | 0 | 0 | 0 | 0 | 0.0 |
| Total | 0 | 0 | 0 | 0 | 0 | 0 | 0 | 0 | 1 | 0 | 0 | 0 | 0.1 |

| **Proximal clone_Cut-GOF (7 cell distance onward from tip)** | | | | | | |
| --- | --- | --- | --- | --- | --- | --- |
| **Directional area** | **# cytonemes with length 0-15μm** | | | | | **Average** |
| 0-30 | 1 | 1 | 1 | 1 | 3 | 1.4 |
| 30-60 | 0 | 3 | 1 | 1 | 0 | 1 |
| 60-90 | 0 | 0 | 2 | 1 | 0 | 0.6 |
| 90-120 | 2 | 0 | 4 | 1 | 0 | 1.4 |
| 120-150 | 0 | 1 | 1 | 0 | 0 | 0.4 |
| 150-180 | 0 | 0 | 1 | 1 | 0 | 0.4 |
| 180-210 | 3 | 0 | 1 | 1 | 1 | 1.2 |
| 210-240 | 1 | 0 | 0 | 1 | 0 | 0.4 |
| 240-270 | 0 | 0 | 1 | 0 | 2 | 0.6 |
| 270-300 | 0 | 0 | 1 | 1 | 3 | 1 |
| 300-330 | 0 | 0 | 0 | 0 | 0 | 0 |
| 330-360 | 2 | 0 | 0 | 0 | 1 | 0.6 |
| Total | 9 | 5 | 13 | 8 | 10 | 9 |
| **Directional area** | **# cytonemes with length 15-30μm** | | | | | **Average** |
| 0-30 | 1 | 0 | 0 | 0 | 0 | 0.2 |
| 30-60 | 0 | 0 | 0 | 0 | 0 | 0 |
| 60-90 | 0 | 0 | 0 | 0 | 0 | 0 |
| 90-120 | 0 | 0 | 0 | 0 | 0 | 0 |
| 120-150 | 0 | 0 | 0 | 0 | 0 | 0 |
| 150-180 | 0 | 0 | 0 | 0 | 0 | 0 |
| 180-210 | 0 | 0 | 0 | 0 | 0 | 0 |
| 210-240 | 0 | 0 | 0 | 0 | 0 | 0 |
| 240-270 | 0 | 0 | 0 | 0 | 0 | 0 |
| 270-300 | 0 | 0 | 0 | 0 | 0 | 0 |
| 300-330 | 0 | 0 | 0 | 0 | 0 | 0 |
| 330-360 | 0 | 0 | 0 | 0 | 0 | 0 |
| Total | 1 | 0 | 0 | 0 | 0 | 0.2 |
| **Directional area** | **# cytonemes with length >30μm** | | | | | **Average** |
| 0-30 | 0 | 0 | 0 | 0 | 0 | 0 |
| 30-60 | 0 | 0 | 0 | 0 | 0 | 0 |
| 60-90 | 0 | 0 | 0 | 0 | 0 | 0 |
| 90-120 | 0 | 0 | 0 | 0 | 0 | 0 |
| 120-150 | 0 | 0 | 0 | 0 | 0 | 0 |
| 150-180 | 0 | 0 | 0 | 0 | 0 | 0 |
| 180-210 | 0 | 0 | 0 | 0 | 0 | 0 |
| 210-240 | 0 | 0 | 0 | 0 | 0 | 0 |
| 240-270 | 0 | 0 | 0 | 0 | 0 | 0 |
| 270-300 | 0 | 0 | 0 | 0 | 0 | 0 |
| 300-330 | 0 | 0 | 0 | 0 | 0 | 0 |
| 330-360 | 0 | 0 | 0 | 0 | 0 | 0 |
| Total | 0 | 0 | 0 | 0 | 0 | 0 |
