# Supplementary material for: Feedback regulation of cytoneme-mediated transport shapes a tissue-specific FGF morphogen gradient
Source: eLife. 2018 Oct 17;7:e38137. doi: 10.7554/eLife.38137 (PMC6224196; doi:10.7554/eLife.38137)
Supplement: Figure 7—source data 5. [file elife-38137-fig7-data5.docx]

**Number of ASP cytonemes oriented in different directions from *yan* GOF clones.**

| **Distal clone_Yan (1-6 cell distance from tip)** | | | | | | | | | | | |
| --- | --- | --- | --- | --- | --- | --- | --- | --- | --- | --- | --- |
| **Directional area** | **# cytonemes with length 0-15μm** | | | | | | | | | | **Average** |
| 0-30 | 1 | 1 | 1 | 2 | 0 | 2 | 2 | 0 | 0 | 1 | 1.0 |
| 30-60 | 2 | 2 | 0 | 1 | 0 | 1 | 2 | 0 | 2 | 1 | 1.1 |
| 60-90 | 1 | 3 | 0 | 1 | 0 | 0 | 0 | 0 | 0 | 1 | 0.6 |
| 90-120 | 0 | 3 | 1 | 1 | 0 | 0 | 0 | 0 | 0 | 0 | 0.5 |
| 120-150 | 0 | 0 | 0 | 0 | 0 | 0 | 0 | 0 | 0 | 0 | 0.0 |
| 150-180 | 0 | 0 | 1 | 0 | 0 | 1 | 0 | 0 | 0 | 0 | 0.2 |
| 180-210 | 0 | 0 | 2 | 0 | 2 | 1 | 1 | 1 | 0 | 0 | 0.7 |
| 210-240 | 0 | 0 | 2 | 0 | 0 | 1 | 0 | 0 | 0 | 0 | 0.3 |
| 240-270 | 1 | 0 | 2 | 2 | 0 | 0 | 0 | 4 | 0 | 0 | 0.9 |
| 270-300 | 0 | 1 | 3 | 1 | 0 | 1 | 0 | 2 | 0 | 1 | 0.9 |
| 300-330 | 1 | 1 | 2 | 0 | 0 | 0 | 1 | 2 | 0 | 1 | 0.8 |
| 330-360 | 0 | 3 | 5 | 4 | 1 | 0 | 2 | 1 | 1 | 2 | 1.9 |
| SUM | 6 | 14 | 19 | 12 | 3 | 7 | 8 | 10 | 3 | 7 | **8.9** |
| **Directional area** | **# cytonemes with length 15-30μm** | | | | | | | | | | **Average** |
| 0-30 | 0 | 0 | 0 | 0 | 1 | 0 | 0 | 0 | 2 | 1 | 0.4 |
| 30-60 | 0 | 0 | 0 | 0 | 0 | 0 | 0 | 0 | 0 | 0 | 0.0 |
| 60-90 | 0 | 0 | 0 | 0 | 0 | 0 | 0 | 0 | 0 | 0 | 0.0 |
| 90-120 | 0 | 0 | 0 | 0 | 0 | 0 | 0 | 0 | 0 | 0 | 0.0 |
| 120-150 | 0 | 0 | 0 | 0 | 0 | 0 | 0 | 0 | 0 | 0 | 0.0 |
| 150-180 | 0 | 0 | 0 | 0 | 0 | 0 | 0 | 0 | 0 | 0 | 0.0 |
| 180-210 | 0 | 0 | 0 | 0 | 0 | 0 | 0 | 0 | 0 | 0 | 0.0 |
| 210-240 | 0 | 0 | 0 | 0 | 0 | 0 | 0 | 0 | 0 | 0 | 0.0 |
| 240-270 | 0 | 0 | 0 | 0 | 0 | 0 | 0 | 0 | 0 | 0 | 0.0 |
| 270-300 | 0 | 2 | 0 | 0 | 0 | 0 | 0 | 0 | 0 | 0 | 0.2 |
| 300-330 | 0 | 1 | 0 | 0 | 0 | 0 | 0 | 0 | 0 | 0 | 0.1 |
| 330-360 | 0 | 0 | 0 | 0 | 0 | 1 | 0 | 1 | 2 | 1 | 0.5 |
| SUM | 0 | 3 | 0 | 0 | 1 | 1 | 0 | 1 | 4 | 2 | **1.2** |
| **Directional area** | **# cytonemes with length > 30μm** | | | | | | | | | | **Average** |
| 0-30 | 0 | 0 | 0 | 0 | 0 | 0 | 0 | 0 | 0 | 0 | 0.0 |
| 30-60 | 0 | 0 | 0 | 0 | 0 | 0 | 0 | 0 | 0 | 0 | 0.0 |
| 60-90 | 0 | 0 | 0 | 0 | 0 | 0 | 0 | 0 | 0 | 0 | 0.0 |
| 90-120 | 0 | 0 | 0 | 0 | 0 | 0 | 0 | 0 | 0 | 0 | 0.0 |
| 120-150 | 0 | 0 | 0 | 0 | 0 | 0 | 0 | 0 | 0 | 0 | 0.0 |
| 150-180 | 0 | 0 | 0 | 0 | 0 | 0 | 0 | 0 | 0 | 0 | 0.0 |
| 180-210 | 0 | 0 | 0 | 0 | 0 | 0 | 0 | 0 | 0 | 0 | 0.0 |
| 210-240 | 0 | 0 | 0 | 0 | 0 | 0 | 0 | 0 | 0 | 0 | 0.0 |
| 240-270 | 0 | 0 | 0 | 0 | 0 | 0 | 0 | 0 | 0 | 0 | 0.0 |
| 270-300 | 0 | 0 | 0 | 0 | 0 | 0 | 0 | 0 | 0 | 0 | 0.0 |
| 300-330 | 0 | 0 | 0 | 0 | 0 | 0 | 0 | 0 | 0 | 0 | 0.0 |
| 330-360 | 0 | 0 | 0 | 0 | 0 | 0 | 0 | 0 | 0 | 0 | 0.0 |
| SUM | 0 | 0 | 0 | 0 | 0 | 0 | 0 | 0 | 0 | 0 | **0.0** |

| **Proximal clone_Yan (7 cell distance onward from tip)** | | | | | | |
| --- | --- | --- | --- | --- | --- | --- |
| **Directional area** | **# cytonemes with length 0-15μm** | | | | | **Average** |
| 0-30 | 2 | 3 | 1 | 2 | 2 | 2 |
| 30-60 | 1 | 3 | 2 | 2 | 2 | 2 |
| 60-90 | 0 | 2 | 0 | 4 | 2 | 1.6 |
| 90-120 | 0 | 2 | 1 | 2 | 0 | 1 |
| 120-150 | 2 | 0 | 0 | 0 | 0 | 0.4 |
| 150-180 | 2 | 0 | 0 | 1 | 0 | 0.6 |
| 180-210 | 0 | 1 | 0 | 2 | 0 | 0.6 |
| 210-240 | 0 | 2 | 0 | 2 | 0 | 0.8 |
| 240-270 | 0 | 2 | 3 | 2 | 3 | 2 |
| 270-300 | 2 | 1 | 7 | 2 | 3 | 3 |
| 300-330 | 0 | 1 | 2 | 0 | 1 | 0.8 |
| 330-360 | 3 | 4 | 2 | 3 | 2 | 2.8 |
| SUM | **12** | 21 | 18 | 22 | 15 | **17.6** |
| **Directional area** | **# cytonemes with length 15-30μm** | | | | | **Average** |
| 0-30 | 0 | 0 | 0 | 0 | 0 | 0 |
| 30-60 | 0 | 0 | 0 | 0 | 0 | 0 |
| 60-90 | 0 | 0 | 0 | 0 | 0 | 0 |
| 90-120 | 0 | 0 | 0 | 0 | 0 | 0 |
| 120-150 | 0 | 0 | 0 | 0 | 0 | 0 |
| 150-180 | 0 | 0 | 0 | 0 | 0 | 0 |
| 180-210 | 0 | 0 | 0 | 0 | 0 | 0 |
| 210-240 | 0 | 0 | 0 | 0 | 0 | 0 |
| 240-270 | 0 | 0 | 0 | 0 | 0 | 0 |
| 270-300 | 0 | 0 | 0 | 0 | 0 | 0 |
| 300-330 | 0 | 0 | 0 | 0 | 0 | 0 |
| 330-360 | 0 | 0 | 0 | 0 | 0 | 0 |
| SUM | **0** | 0 | 0 | 0 | 0 | **0** |
| **Directional area** | **# cytonemes with length > 30μm** | | | | | **Average** |
| 0-30 | 0 | 0 | 0 | 0 | 0 | 0 |
| 30-60 | 0 | 0 | 0 | 0 | 0 | 0 |
| 60-90 | 0 | 0 | 0 | 0 | 0 | 0 |
| 90-120 | 0 | 0 | 0 | 0 | 0 | 0 |
| 120-150 | 0 | 0 | 0 | 0 | 0 | 0 |
| 150-180 | 0 | 0 | 0 | 0 | 0 | 0 |
| 180-210 | 0 | 0 | 0 | 0 | 0 | 0 |
| 210-240 | 0 | 0 | 0 | 0 | 0 | 0 |
| 240-270 | 0 | 0 | 0 | 0 | 0 | 0 |
| 270-300 | 0 | 0 | 0 | 0 | 0 | 0 |
| 300-330 | 0 | 0 | 0 | 0 | 0 | 0 |
| 330-360 | 0 | 0 | 0 | 0 | 0 | 0 |
| SUM | 0 | 0 | 0 | 0 | 0 | **0** |
