# Supplementary material for: Feedback regulation of cytoneme-mediated transport shapes a tissue-specific FGF morphogen gradient
Source: eLife. 2018 Oct 17;7:e38137. doi: 10.7554/eLife.38137 (PMC6224196; doi:10.7554/eLife.38137)
Supplement: Source code 1. [file elife-38137-code1.docx]

library(ggplot2)

filename="Proximal clone_WT"

file_t= "FinalData\\"

file<-paste(file_t,filename,".csv",sep="")

#data<-read.csv("FinalData\\Proximal clone_WT.csv",head=TRUE,sep=",")

data<-read.csv(file,head=TRUE,sep=",")

cols<-c("blue","yellow","red")

p<-ggplot(data, aes(x=data$Range,y=data$Counts,fill=data$Length)) +

#theme_bw()+

#theme_minimal() +

geom_bar(width = 30, colour="black", stat="identity") +

#geom_hline(yintercept = 2.5) +

geom_vline(xintercept = c(0,90,180,270)) +

#scale_fill_manual(values = cols) +

#scale_y_discrete(drop = FALSE) +

theme(legend.box.just = "top",legend.position = "bottom") +

theme(panel.grid.major = element_line(colour = "gray"),

panel.grid.minor = element_line(colour = "blue"),

panel.background = element_blank(),

axis.line = element_line(colour = "black"))+

labs(title = filename,

fill = "Length range(um)",

y = "Cytoneme number", limits = c(0, 100),colour = "Cylinders") +

coord_polar(theta = "x", start=-pi/2, direction=-1) +

scale_x_discrete("", limits = c(0,90,180,270), labels = c(0,90,180,270))

#scale_y_continuous(limits=c(0, 6), breaks= c(1:6))

p
